# Dihydropyridines’ metabolites-induced early apoptosis after myocardial infarction in rats; new outlook on preclinical study with M-2 and M-3

**DOI:** 10.1007/s10495-015-1205-2

**Published:** 2015-12-14

**Authors:** Katarzyna A. Mitręga, Jerzy Nożyński, Maurycy Porc, Adrianna M. Spałek, Tadeusz F. Krzemiński

**Affiliations:** Chair and Department of Pharmacology, Medical University of Silesia, ul. Jordana 19, 41-808 Zabrze, Poland; Silesian Centre for Heart Diseases, ul. Szpitalna 2, 41-800 Zabrze, Poland

**Keywords:** Furnidipines’ metabolites, Reperfused myocardial infarction, Programmed cell death, Rat, Hemodynamic, Arrhythmias

## Abstract

Our previous studies established cardio-protective effects of furnidipine and its active metabolites called M-2 and M-3. The aim of current research was to compare the effects of single oral pretreatment with 20 mg kg^−1^ of M-2 and M-3 on mortality, different forms of arrhythmias, blood pressures parameters and ST-segment changes during occlusion (for 90 min) and reperfusion in the model of myocardial infarction in rats evoked by left anterior descending coronary artery occlusion. Additionally, the development of programmed cell death and biochemical parameters in blood serum were studied at 4th day after infarction. Furnidipines’ metabolites effectively reduced mortality index while did not markedly influence on blood pressures parameters, arrhythmias, ST-segment changes as well as biochemical parameters. Intriguingly, programmed cell death study (TUNEL) showed distinct increase in the amount of apoptotic nuclei in post-infarcted myocardium, granulation tissue and what is more in arteriolar walls after M-2 and M-3 application. Moreover, M-2 turned out to be more powerful in stimulation of apoptosis in granulation tissue surrounding infarcted area whereas M-3 presented balanced profile in this matter. Taking into account that programmed cell death plays positive role in post-infarcted heart healing, M-2 presents itself as more attractive agent for oral pretreatment in early stages of ischemia by non-stable individuals due to its more specific action in stimulation repairing processes in granulation tissue as well as in arteriolar walls. While M-2 and M-3 are common metabolites present in degradation pathways of many widely used dihydropyridines in clinic, this key fact put the new outlook on understanding additional mechanism and effects of not only furnidipines’ metabolites but also other dihydropyridines.

## Introduction

Although reperfusion of the myocardium following coronary occlusion reduces infarct size and improves its function [1, 2], numerous studies point to potential detrimental effect of reperfusion on endothelium, myocardial muscle structure, coronary vascular reactivity and potentially lethal rhythm disturbances [3–5] as well as for apoptosis [6, 7]. In addition, observations made during therapeutic revascularization procedures in infarct patients strongly suggest that reperfusion might facilitate remobilization of small vessels [8–10]. Despite considerable efforts, results in protection or therapeutic management of reperfusion-triggered pathologies and ischemic coronary artery diseases still are far from being satisfactory.

The dihydropyridine derivatives (DHPs) currently used for therapeutic purposes possess L-type calcium channel blocking properties, and treatment of hypertension and certain specific forms of angina pectoris remain to be their main therapeutic indications [11]. In addition, it has been shown that DHPs can protect the heart from stunning, ischemia as well as ventricular arrhythmias, and that they possess beneficial effects against experimental atherosclerosis [9, 10, 12–19]. Furthermore, many studies revealed that calcium channel blockers can reduce infarct size in experimental animals [5, 20–22]. It is unclear though, whether these therapeutically interesting pharmacological properties demonstrated for many DHPs are only due to their blocking effects on the L-type calcium channel. In fact, it is well established that some of them can enhance opening probability of L-type channels [23] and yet others can effectively modulate various ion channels and pharmacological targets as well. Due to their different activities, DHPs are sometimes referred to be pharmacologically “privileged structures” [24, 25].

**Fig. 1:**
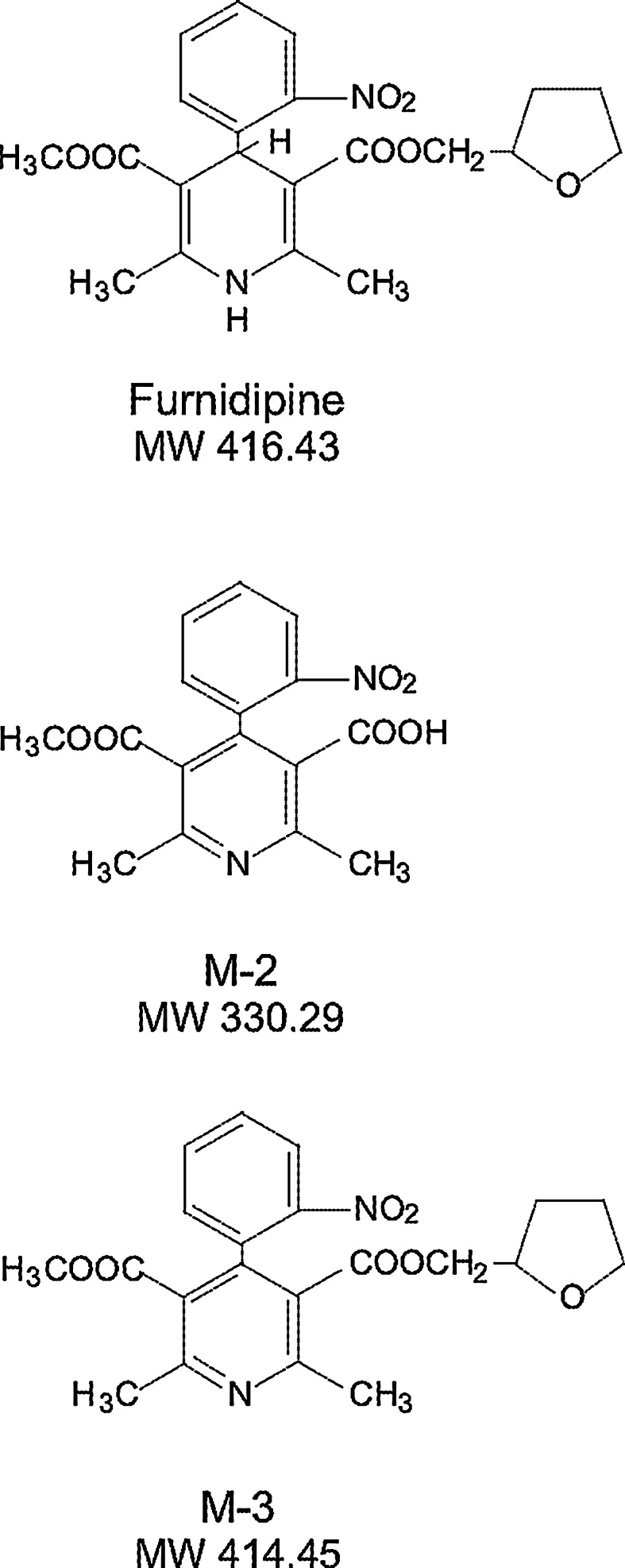
Structure of parent drug, furnidipine, and its metabolites M-2 and M-3

Attempts to reach such goals have led to the identification of furnidipine (FUR) [26, 27]. Initially, FUR (for structure see Fig. 1) was identified structurally and functionally as a DHP with potent L-type calcium channel blocking activity, good oral bio-availability and exceptionally high safety margin in pre-clinical studies [28, 29]. Later efforts to define its pharmacological activity profile revealed though, that unlike most therapeutically used calcium antagonists, FUR is highly selective in relaxing both venous capacitance and arterial vessels resistance. Although it has no influence on heart conduction system, it revealed antiarrhythmic activity in the aconitine-induced arrhythmias model in rats [26, 27, 29]. In addition, FUR pretreatment afforded protection against norepinephrine-induced cardiomyocyte necrosis in rats and its effective dose range in this model was much broader than that of nitrendipine. What is more, after oral as well as intravenous pretreatment, FUR can not only dose-dependently influence on arterial blood pressure through the vessels relaxation and reduction in myocardial oxygen consumption, but also can afford protection against reperfusion-triggered myocardial damages and especially rats’ mortality decrease evoked by lethal arrhythmias [30]. Interestingly, its protective effects on myocardial tissue damage, judged by the creatine kinase reduction in blood, were observed as well [30]. Although antihypertensive effects of FUR were apparent in several animal models [26, 31] results of various controlled clinical trials indicated that in comparison to several other calcium antagonists, its antihypertensive efficacy is negligible.

At least two FUR metabolites lacking calcium channel blocking activity with prolonged plasma half-life have already been identified in human volunteers and called as M-2 and M-3 [31]. In addition, plasma levels of FUR oxidative metabolites were always higher than that of the parent molecule after oral administration. Consequently, both metabolites were screened for their typical blocking activity and cardio-protective potential in several in vitro models commonly used for such purposes [31]. Early data in our laboratories have revealed similar efficacy of M-2 in the same experimental model as used for FUR. Unlike furnidipine, M-2 did not modulate blood pressure parameters or heart rate [30]. Furthermore, other authors proved that M-2 itself did not influence significantly the guinea pig cardiomyocyte action potential. However, it antagonized dose-dependently (1 × 10^−7^–3 × 10^−7^ M) and markedly the veratridine-induced action potential lengthening as well as the anoxia-induced action potential shortening and additionally, prevented from the cellular shape changes [31].

Comparing the influence of continuous infusion of pro-drug to M-2 and M-3 in working rats’ heart model [32–34]. We established that FUR evoked significantly weaker influence on coronary and aortic flow, whereas both metabolites caused a significant coronary flow increase. What is more, M-3 caused the aortic systolic and diastolic pressures decrease. Due to clear differences found between all three agents, we concluded that the cardio-depressant potency of both metabolites is overcome by advantageous vasodilatatory effect. In addition, studying the effects of M-2 in two different models of working heart ischemia (low-flow and regional), we confirmed that it improved coronary flow in both models, while favorably maintaining aortic pressure parameters [35]. Moreover, it provided outstanding protection against deleterious effects of calcium overload (induced by veratridine in the Langendorff heart) by significant prevention of the left ventricular diastolic pressure rise and coronary flow decrease [36, 37].

Concluding these results, it allowed us to propose the working hypothesis that the protective effects against cellular damage evoked by FUR could be mainly attributed to its metabolites and moreover, indicate other sites of action different from the L-type calcium channel suggesting pleiotropic effects on the ischemic heart by imparting protection in various ways [34, 35, 38].

Since in vitro results do not always correspond to in vivo outcomes, we studied the influence of M-2 on hemodynamic parameters and ischemia- and reperfusion-induced arrhythmias in rats [15, 30, 39] for further testing its potential value as a therapeutic agent in infarcted hearts. Dose- and time-response curves were obtained after oral administration of M-2 in order to establish its pharmacokinetic properties and pharmacodynamic half-life. We proved that it significantly reduced mortality, incidence and duration of severe arrhythmias with differential influence on blood pressure, which depended on dose and time of administration [35]. The optimal oral dose of M-2 and M-3 was 20 mg kg^−1^ and those has been used in current study as well.

Considering the all promising results, we decided to perform present study in order to compare the effects of M-2 as well as M-3 pretreatment on different forms of arrhythmias, blood pressures and ST-segment changes during occlusion (for 90 min) and reperfusion in the model of myocardial infarction in rats evoked by permanent left anterior descending coronary artery (LAD) occlusion [40]. Furthermore, the development of programmed cells death and biochemical parameters in blood serum for the heart damage were studied at 4th day after myocardial infarction.

## Materials and methods

### Experimental animals

Male *Sprague*–*Dawley* rats (*n* = 54; Central Animal Farm, Medical University of Silesia, Katowice, Poland) weighting approx. 320 ± 25 g and maintained under standard condition (ambient temperature 21–23 °C; with 12 h dark/light cycle) with ad libitum access to food (Altromin 1220, Altromin GmbH, Lage, Germany) and tap water, served as experimental animals. The animals were fasted overnight before the experiment. The study was performed with the approval of the Local Bioethical Committee and all experiments were conducted in accordance with NIH regulations of animals care described in the “Guide for the Care and Use of Laboratory Animals” (NIH publication, p. 2–107, revised 1996).

### Drugs and reagents used

Two metabolites of furnidipine were used: M-2 [2,6-dimethyl-5methoxy-carbonyl-4-(2′-nitrophenyl)-pyridine-3-carboxyliquide acid, MW 330.29] and M-3 [3-methyl 5-(tetrahydrofuran-2-yl) methyl 2,6-dimethyl-4-(2′-nitrophenyl) pyridine-3,5-dicarboxylate, MW 414.45] (Fig. 1). Both metabolites were supplied by Cermol S.A. (Geneva, Switzerland) and were primarily dissolved in diluted dimethylsulfoxide (DMSO) and later in water. For oral administration, M-2 or M-3 solutions were prepared in 0.4 % aqueous dimethylsulfoxide and given in a volume of 5 mL kg^−1^ through inserted stomach-tube. Water was used in the control group, nevertheless, in order to consider the influence of DMSO itself, additional control group was added (0.4 % DMSO). Unless otherwise stated, all other reagents were of the highest purity and were supplied by Sigma Chemical Co. (Deisenhofen, Germany).

### Experimental infarction in rats

For this study an improved preparation previously described by others [41, 42] with own modifications described in details elsewhere [32, 40, 43–46] was used. The Lambeth Conventions were used also as a guideline for this research [47].

The rats were anesthetized with pentobarbital (60 mg kg^−1^/at the beginning/+30 mg kg^−1^ i.p./10 min before reperfusion/pentobarbital sodium salt Sigma, Deisenhofen, Germany). To compare the depth of anesthesia, reflex response to noise and pain induced by the pinching of the limbs and distal portion of the tail, were tested in each rat at the beginning and the end of the experiment as prescribed [48, 49]. Rectal temperature was maintained at approximately 38 °C.

The left common carotid artery was cannulated with a filled catheter (saline with 2 IU mL^−1^ heparin) for systolic and diastolic blood pressures (BPs, BPd) measurement using a ISOTEC transducer (Hugo Sachs Elektronik, March-Hugstetten, Germany).

The myocardial infarction was induced by permanent left anterior descending coronary artery occlusion for 90 min and followed by 15 min of reperfusion. Only rats that survived until 4 day after infarction were taken into account during our investigations.

In brief, the trachea was incised longitudinally and cannulated to allow artificial ventilation. The chest was opened under ventilation with room air (55–60 % humidity, 23 °C, stroke volume 0.8 mL 100 g^−1^ of body weight; rate 54 strokes min with the positive end-respiratory pressure of 1 cm H_2_O; Rodent VENTILATOR-UB 7025, Hugo Sachs Elektronik, March-Hugstetten, Germany) [50] by left thoracotomy at the fifth intercostal space and the fifth and fourth ribs were sectioned approximately 2 mm from the left margin of the sternum. After opening the pericardium the heart was not exteriorized and a sling (6/0 Prolene 0.7 suture attached to 3/8 circled BV-1 a 9.3 mm atraumatic, reverse cutting needle, EH 7406H, Ethicon GmbH, Norderstedt, Germany) was placed around LAD close to its origin (2 mm below). Then the ligature was passed through a plastic pad (polyethylene, 2 mm OD/0.5 ID, thickness 0.2 mm). The left coronary artery was occluded by applying tension to the ligature while pressing the pad onto the heart surface. Tension was maintained by clamping a climb clip (Titan climb clip, LT-100, Ethicon). Successful occlusion was immediately confirmed by ischemia-induced alteration in ECG (ST-elevation e.g.) and observation of an arising pale ischemic zone below the climb clip.

The ECG was picked up from standard limb leads using needle electrodes and recorded synchronously with blood pressure curve on high-speed chart recorders (ECG thermo recorder E-30, Farum, Poznań, Poland; 100 mm s^−1^ and Line Recorder TZ 4620, Laboratorni Pristroje, Praha, Czech, respectively) and displayed parallely on a digital cardiomonitor (CMK 405, TEMED, Zabrze, Poland) throughout the experiment.

Independently, all received signals (BP and ECG) were transmitted through 16-channel A/D converter and stored away by IBM compatible computer with the necessary own software for data acquisition and elaboration (off-line).

At the end of the experiment (105 min), tissues were sutured in layers (4-0 Deklene TM-II, 1.5, D-5427, Ethicon) excluding pericardium (avoiding heart tamponage). The rats awaked in few hours after closing the thorax. Furthermore, the rats which survived with myocardial infarction were housed for next 4 days.

After this 4 days the rats were again anaesthetized with pentobarbital (60 mg kg^−1^, i.p.). In the external jugular vein, a PE 50 catheter was placed i.v. for the injection of the Evan’s blue dye (to confirm successful reperfusion) followed by pentobarbital for lethal anesthesia at the end of the experiment.

The trachea was cannulated to allow artificial ventilation with room air (Rodent VENTILATOR-UB 7025, stroke volume 0.8 mL 100 g^−1^ body weight and rate 54 strokes min^−1^, Hugo Sachs Elektronik, March-Hugstetten, Germany). The chest was opened by a left thoracotomy, the rats’ blood from aortic arch was collected for further biochemical estimations and the heart was subsequently excised in order to determinate myocardial infarct size as well as saved in formalin (less than 7 days) for the routine histological study and apoptosis (TUNEL) procedure described below.

### Experimental groups, design and measured parameters

The rats were randomly divided into four groups. Same doses of M-2 and M-3 (20 mg kg^−1^ each) or the vehicles (water or 0.4 % DMSO in the volume of 5 mL kg^−1^ each) were orally administered 1 h before LAD occlusion. At the beginning of our study, each experimental group consisted of: water (*n* = 7), DMSO (*n* = 13), M-2 (*n* = 12) and M-3 (*n* = 12) rats. The LAD was occluded for 90 min and then reopened for 15 min. The mortality index (MI) was calculated for the rats that survived this period (Table 1).

**Table 1:** Effects of single oral pretreatment with 20 mg kg^−1^ of M-2 or M-3 on blood pressure during 90 min of the left anterior coronary occlusion and 15 min of reperfusion in rats

Experimental group	Continuous blood pressure measurement (mmHg) (mean ± SD)										
	Occlusion (90 min)									Reperfusion (15 min)	
	5 min	15 min	25 min	35 min	45 min	55 min	65 min	75 min	85 min	95 min	105 min
	BPs/BPd	BPs/BPd	BPs/BPd	BPs/BPd	BPs/BPd	BPs/BPd	BPs/BPd	BPs/BPd	BPs/BPd	BPs/BPd	BPs/BPd
Control *n* = 4/7 [MI 42.8 %]	142 ± 10 116 ± 9.1	141 ± 33 114 ± 25	124 ± 31 100 ± 27	123 ± 41 92 ± 28	122 ± 46 97 ± 39	133 ± 38 98 ± 43	133 ± 38 107 ± 35	115 ± 23 80 ± 11	113 ± 19 83 ± 14	140 ± 26 157 ± 51	139 ± 13 107 ± 6
DMSO *n* = 10/13 [MI 23.1 %]	140 ± 17 114 ± 16	137 ± 28 111 ± 24	138 ± 30 111 ± 30	130 ± 38 103 ± 38	121 ± 36 94 ± 38	121 ± 30 95 ± 32	124 ± 31 96 ± 35	129 ± 33 102 ± 36	130 ± 28 103 ± 31	113 ± 16 86 ± 19	115 ± 21 87 ± 26
M-2 *n* = 10/12 [MI 16.6 %]	138 ± 14 111 ± 14	144 ± 17 116 ± 14	136 ± 22 110 ± 19	136 ± 16 111 ± 15	130 ± 22 103 ± 20	121 ± 27 95 ± 23	124 ± 23 96 ± 17	124 ± 22 97 ± 17	121 ± 23 96 ± 18	115 ± 22 91 ± 20	124 ± 26 96 ± 21
M-3 *n* = 10/12 [MI 16.6 %]	146 ± 20 117 ± 16	147 ± 10 118 ± 6	145 ± 17 113 ± 16	138 ± 19 108 ± 20	131 ± 19 99 ± 21	133 ± 14 100 ± 16	129 ± 18 98 ± 19	123 ± 29 95 ± 29	126 ± 29 99 ± 30	124 ± 16 97 ± 20	127 ± 20 98 ± 25

In the first column the data indicates number of rats that survived/number of animals in the group at the start of the experiment and in parentheses mortality index (MI) was given. The differences in BP values between the groups [systolic (BPS) and diastolic (BPd)] were calculated using non-parametric Kruskal–Wallis ANOVA test with appropriate post hoc test

The continuous blood pressure parameters (BPs and BPd) measurements were performed and recorded every 10 min during the occlusion and reperfusion (from 5th to 105th min; see Table 1). The ST-segment changes (in mm) were estimated every 5 min during the occlusion and reperfusion periods (see Table 2).

**Table 2:** Effects of single oral pretreatment with 20 mg kg^−1^ of M-2 or M-3 on ST-segment changes during 90 min of left anterior coronary occlusion and 15 min of reperfusion in rats

Experimental group	ST-segment estimation (J point (mm)) (mean ± SD)										
	Before	Occlusion									
	1 min	5 min	10 min	20 min	30 min	40 min	50 min	60 min	70 min	80 min	90 min
Control *n* = 4/7	−0.05 ± 0.06	−0.027 ± 0.105	−0.022 ± 0.021	0.132 ± 0.204	0.092 ± 0.147	0.082 ± 0.159	0.07 ± 0.120	0.01 ± 0.145	0.055 ± 0.151	0.085 ± 0.179	0.11 ± 0.16
DMSO *n* = 10/13	−0.021 ± 0.044	−0.02 ± 0.042	−0.009 ± 0.028	0.082 ± 0.109	0.083 ± 0.101	0.073 ± 0.026	0.073 ± 0.083	0.079 ± 0.099	0.079 ± 0.099	0.064 ± 0.091	0.051 ± 0.055
M-2 *n* = 10/12	−0.015 ± 0.03	−0.006 ± 0.07	−0.007 ± 0.01	0.086 ± 0.1	0.095 ± 0.11	0.09 ± 0.11	0.101 ± 0.13	0.119 ± 0.15	0.114 ± 0.15	0.096 ± 0.15	0.096 ± 0.12
M-3 *n* = 10/12	−0.041 ± 0.047	−0.02 ± 0.043	0.0 ± 0.062	0.061 ± 0.125	0.158 ± 0.318	0.067 ± 0.12	0.076 ± 0.042	0.081 ± 0.134	0.07 ± 0.122	0.073 ± 0.117	0.088 ± 0.118

Experimental group	ST-segment estimation (J point (mm)) (mean ± SD)										
	Reperfusion										
	100 min	105 min									
Control *n* = 4/7	0.095 ± 0.119	0.075 ± 0.116									
DMSO *n* = 10/13	0.05 ± 0.073	0.047 ± 0.074									
M-2 *n* = 10/12	0.045 ± 0.09	0.042 ± 0.09									
M-3 *n* = 10/12	0.066 ± 0.148	0.06 ± 0.141									

The differences in ST-segment changes was calculated using non-parametric Kruskal–Wallis ANOVA test with appropriate post hoc test. For other details see Table 1 and text

**Fig. 2:**
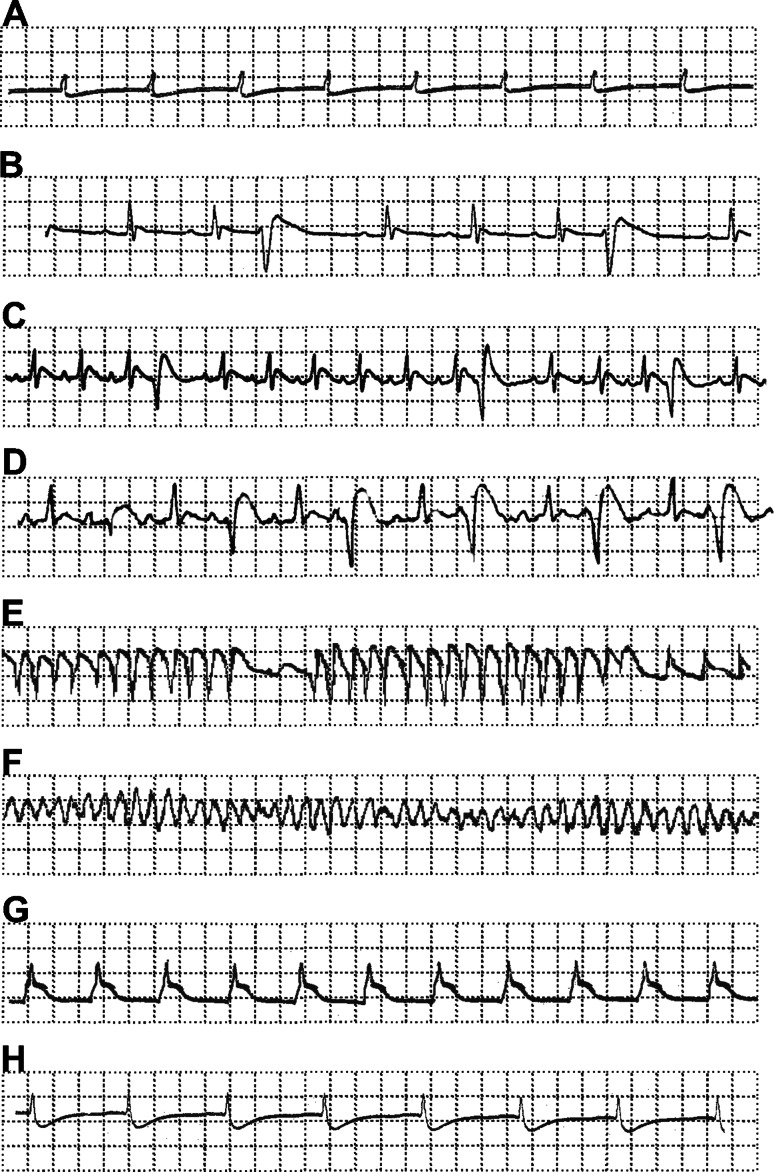
Characteristic electrocardiogram tracings (recorded from I limb lead with recorder speed 100 mm s^−1^): *a* normal tracing before left anterior descending coronary artery (LAD) occlusion, *b* ventricular extrasystoly (VE), *c* multiple premature ventricular beats (PVBs), *d* bigeminy, *e* ventricular tachycardia (VT), *f* ventricular fibrillation (VF), *g* ST-segment elevation during ischemia/observed after LAD occlusion/, *h* ECG tracing during late reperfusion

The number of the premature ventricular beats (PVBs) during occlusion and reperfusion was counted as well as the incidence (in %) and duration (in seconds) of the spontaneously reversible ventricular fibrillation (VF), ventricular tachycardia (VT), salvos, bigeminy or trigeminy that occurred during both periods were measured from the continuous ECG recordings using own software (off-line) (see Table 3). All rhythms’ disturbances in rats were distinguished due to rules described in details elsewhere [47, 51] (Fig. 2).

**Table 3:** Effects of single oral pretreatment with 20 mg kg^−1^ of M-2 or M-3 on different forms of arrhythmias occurred during 90 min of left anterior coronary occlusion and 15 min of reperfusion in rats

Experimental group	Arrhythmias calculation (mean ± SD)											
	Occlusion (90 min)						Reperfusion (15 min)					
	PVBs (number)	VT duration (s)	VF duration (s)	Salvos duration (s)	Bigeminy duration (s)	Trigeminy duration (s)	PVBs (number)	VT duration (s)	VF duration (s)	Salvos duration (s)	Bigeminy duration (s)	Trigeminy duration (s)
Control *n* = 4/7	17 ± 18.7*	5.3 ± 10.5	3.75 ± 7.5	2.75 ± 3.2	19.75 ± 25.7	0	0.25 ± 0.5	0	0	0	0	0
DMSO *n* = 10/13	10.8 ± 13.3*	8.1 ± 15.2	9.0 ± 28.5	1.2 ± 2.6	11.3 ± 19.5	2.7 ± 7.5	1.1 ± 3.14	0	0	0	0	0
M-2 *n* = 10/12	7.6 ± 9.2*	17.9 ± 32.64	3.0 ± 9.4	2.1 ± 3.2	25.2 ± 33.7*	10.8 ± 34.15	1.3 ± 2.75	0	0	0	0.6 ± 1.89	1.5 ± 4.74
M-3 *n* = 10/12	8.2 ± 11.5*	23.1 ± 52.8	0.6 ± 1.89	2.4 ± 3.77	18.8 ± 29.5	3.0 ± 9.4	0.6 ± 1.35	0	2.1 ± 6.64	0	0	0

The differences in arrhythmias duration [ventricular tachycardia (VT), ventricular fibrillation (VF), salvos, bigeminy or trigeminy in seconds] and the number of premature ventricular beats (PVBs) were calculated using non-parametric Kruskal–Wallis ANOVA test with appropriate post hoc test

Values marked with * p < 0.05 are significantly different from the values of corresponding parameter in reperfusion. For other details see Table 1 and text

### Biochemical estimation in blood serum

At the 4th day of experiment, 1 mL of rats’ blood was collected directly from aortic arch and without heparinizing dissolved in saline (1/1 vol/vol.) to analyze creatine kinase (CK, U/L; wavelength 340 nm, Reagent-test, Gilford, Ciba-Cornig, Cambridge, MA, USA) [52], glutamate-pyruvate transaminase (GTP, U/L, 340 nm) and glutamate-oxaloacetate transaminase activity (GOT, U/L, 340 nm) in order to estimate heart muscle damage as well as the level of glucose (mg dL^−1^, 340 nm), urea (mg dL^−1^, 530 nm), bilirubin (mg dL^−1^), creatinine (mg dL^−1^, 340 nm) and α-amylase (U/L, 578 nm) spectophotometrically (Specol 220, VEB Carl Zeiss, Jena, Germany) [53] (Table 4). In order to obtain the physiological values of biochemical parameters mentioned above, the intact group (n = 10) was added to the trial.

**Table 4:** Effects of single oral pretreatment with 20 mg kg^−1^ of M-2 or M-3 on blood parameters measured after 90 min of left anterior coronary occlusion and 15 min of reperfusion in rats

Experimental group	Biochemical estimation in rats’ blood serum (mean ± SD)							
	Glucose (mg/dL)	Bilirubin (mg/dL)	Creatinine (mg/dL)	Amylase (U/L)	GTP (U/L)	GOT (U/L)	Creatine kinase (U/L)	Urea (mg/Dl)
Intact *n* = 10	139.6 ± 6.92	<0.5	<0.5	4 323.3 ± 241.9	21.35 ± 1.29	109.8 ± 9.3	216.4 ± 87.7	53.46 ± 2.68
Control *n* = 4/7	137 ± 31.7	<0.5	<0.5	2 975 ± 697.6	24.8 ± 3.86	109.6 ± 10.3*	384.2 ± 106.8	59.5 ± 0.7
DMSO *n* = 10/13	137.9 ± 17.6	<0.5	<0.5	2 996 ± 512	23.1 ± 5.86	84.8 ± 13.5	362.7 ± 105.2*	60.3 ± 6.7
M-2 *n* = 10/12	128.3 ± 20.2	<0.5	<0.5	3 169 ± 1063.8	22 ± 6.53	85.5 ± 24.8	481.2 ± 317.5*	54.6 ± 7.03
M-3 *n* = 10/12	139.1 ± 12	<.5	<0.5	2 779 ± 627.4	20.2 ± 4.8	64.1 ± 18	184.7 ± 88	60 ± 3.82

Bilirubin and creatinine were below detection limit. For other details see Table 1 and text. The differences in blood parameters was calculated using non-parametric Kruskal–Wallis ANOVA test with appropriate post hoc test

Values marked with * p < 0.05 are significantly different from the values of M-3 group

### Determination of myocardial infarct size

After 4 days of the experiment, the heart was removed and perfused 5 min through the cannula inserted into aorta with 1 mL of Evans blue (2 %; perfusion pressure 135 cm H_2_O). Then it was frozen at −20 °C for 5–20 min, cut into 1.5-2.0 mm sagittal sections and immersed in 1 % solution of 2,3,5-triphenyltetrazolium chloride (TTC, Sigma, Poole, UK) in phosphate buffer (20 mM, pH 7.4) at 37 °C for 5–15 min. The white area without Evans blue and TTC was considered as infarcted necrotic myocardium, the blue area- normal myocardium and the red area (stained by TTC)- ischemic myocardium. The myocardium was dissected according to its colors and weighed separately. The percentage ratio of the weight of infarcted necrotic myocardium to that of total ischemic myocardium (infarcted necrotic myocardium and ischemic non-necrotic myocardium) was calculated and designated as the infarct size [54]. The area of infarct in all survived animals (*n* = 34) was 54 ± 6.8 %. All treatments and measurements were performed by an experimenter blind to the treatment group.

### Morphologic examination

The infarct-related areas of the heart tissue were fixed in neutral buffered formaldehyde adjusted to pH 7.4. After routine processing through graded alcohols and xylene, the tissue was embedded in paraffin. Thin paraffin sections of infarct-related areas of each sectioned heart were stained with hematoxylin–eosin and Masson’s trichrome stains for light microscopic histological evaluation.

The next stage after deparaffinization and dehydration was subjected to enzymatic digestion in humid chamber (a Petri dish, 20 cm OD for 5 slides) at 37 °C with proteinase K (final concentration 20 μg/mL in 10 mM/L Tris–HCl buffer; pH 7.4–8.0). Tissue slices were dropped with 50 μL enzyme solution and incubated for 30 min. During the incubation the tissues slices were covered with parafilm.

After incubation the parafilm leaves were removed and the slides were washed twice dropping distilled water 2 × 5 min). Endogenous peroxidase were blocked in using 0.3 % H_2_O_2_ in methanol per 30 min in Coplin jar at the room temperature. Then the slides were washed twice with PBS (phosphate-buffered saline; 2 × 1 min in Coplin jar) and after permeation with 0.1 % Triton X-100 solution in 0.1 % sodium citrate TUNEL incubation was conducted in humid chamber at 37 °C during 30 min accordingly to description for TUNEL kit (No 1 684 817 for In situ Cell Death Detection Kit, POD, Boehringer Ingelheim, Mannheim, Germany).

The sites with UTP binding were labelled using converter POD (anti-fluorescein antibody conjugated with horse radish peroxidase) and the sites with positive reaction were visualised with diaminobenzidine (DAB) procedure. Cellular nuclei were counterstained with hematoxylin and cytoplasm slightly with eosin. Finally the sides were dehydrated and mounted in Canada balsam. The control, positive slides were made from rat prostate at 3rd day after castration.

The stain in the cells labelled by TUNEL technique was visualised as dark brown precipitate (dark cells). These cells were considered as the cells during the programmed cell death (PCD) process.

After histological evaluation, the main striking or representative areas from the tissue were captured with digital camera and written as *.tif files using 24 bit color palette at fixed magnification (150× magnification) using POLYVAR (Reichert-Leica) light microscope.

The quantitative evaluation of TUNEL-positive cells was done using ImmunoRatio software as percentage of DAB stained nuclei to all nuclei in a region of interest at 150×magnification, separately for myocardial muscle and for resorptive granulation tissue in the site of infarcts’ three consecutive areas [55]. All doubtfully stained areas were excluded from calculations and assessment.

### Statistical analysis

Blood pressures parameters (BPs, BPd), the ST-segment changes, all forms of arrhythmias as well as biochemical parameters were measured only in the reperfusion surviving animals. Except for the mortality index, all other results are expressed as mean ± standard deviation (SD). Because the data were not normally distributed, for all comparisons non-parametric Kruskal–Wallis ANOVA test was used [56] with appropriate post hoc test. In order to estimate the significance between mortality and incidence of different forms of arrhythmias, the Chi square-test (χ^2^; Yates) was used in all comparisons.

The quantitative evaluation of TUNEL-positive cells were compared using Kruskal–Wallis test for equal medians and non-parametric ANOVA with Mann–Whitney pairwise test as post hoc verification of significant probability (Statistica v. 10 software). In all cases differences were considered significant at *p* < 0.05.

## Results

### Mortality index, blood pressures parameters and ECG study in occlusion and reperfusion

The mortality index did not differ significantly among studied groups, however, in M-2 or M-3 treated animals only two animals did not survive the experiment (16 %) while in each controls groups three rats died (42.8 and 23.1 %, respectively) (Table 1).

During 105 min of the continuous blood pressure measurement no significant changes were considered in occlusion as well as in reperfusion between studied groups (Table 1). Similarly, no significant changes have been found in ST-segment between studied groups (Table 2).

Unlikely, the number of PVBs was significantly reduced in reperfusion in compare to occlusion period in all groups (*p* < 0.05). In addition, the bigeminy duration in reperfusion was markedly reduced after M-2 pretreatment (*p* < 0.05). Similar action was observed concerning the trigeminy duration, but this effect was not significant (Table 3).

### Biochemical estimation in serum samples

A single administration of water, 0.4 % DMSO, M-2 or M-3 did not significantly influence on the concentration of glucose, amylase, urea and GTP measured in blood serum at 4th day after infarction.

Only M-3 single pretreatment reduced significantly glutamate- oxaloacetate transaminase concentration in comparison to water control group (*p* < 0.05), however, M-2 and DMSO groups slightly diminished this parameters as well (Table 4). Similarly, M-3 strongly reduced creatine kinase concentration in comparison to M-2 and DMSO groups (*p* < 0.05). It should be mentioned that M-2 caused the highest increase of this parameter among all tested groups. The concentrations of bilirubin and creatinine in serum were below the detection limit in all groups (Table 4).

### Morphological study

At 4th day after myocardial infarction, the histological findings in all studied slices were generally similar in treated and non-treated rats. They represented resorptive inflammatory infiltrations inside and granulation tissue (data not shown).

### Programmed cell death

**Fig. 3:**
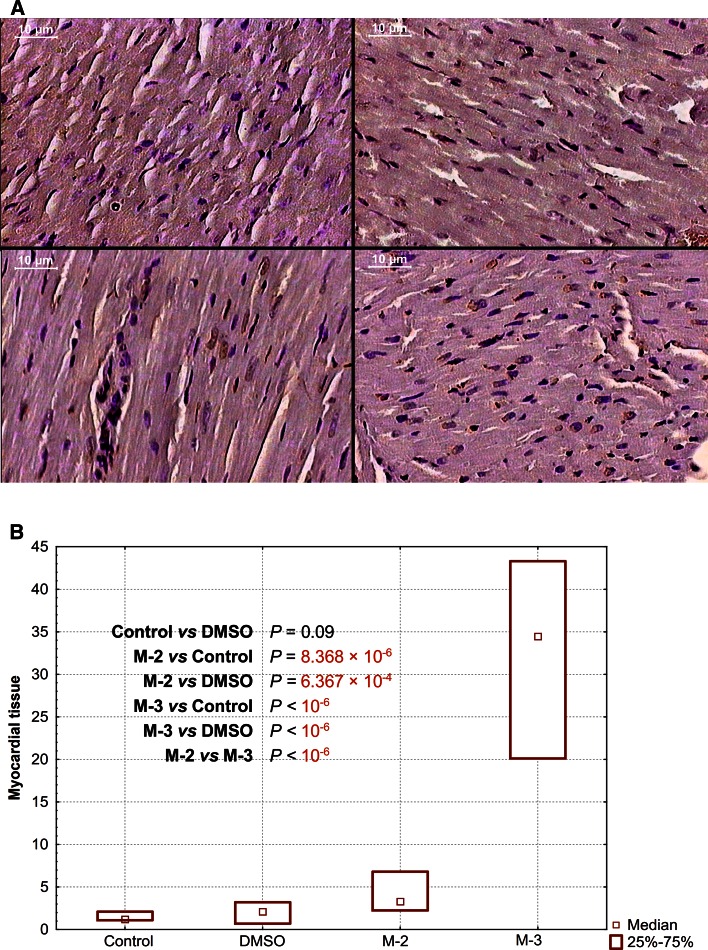
Representative images of apoptotic staining and their quantificative results in rats’ myocardial tissue after single oral pretreatment with 20 mg kg^−1^ of M-2 or M-3. **a** TUNEL reaction. Control (*upper left*): Only one slightly stained brownish cardiocytic nucleus was visible (TUNEL-immunocytochemistry, transmitted light, 100× magnification, *bar* represents 10 µm). DMSO (*upper right*): Scanty positively stained brownish nuclei (TUNEL-positive) and non-apoptotic dark nuclei were visible (TUNEL-immunocytochemistry, transmitted light, 100× magnification, *bar* represents 10 µm). M-2 (*down left*): TUNEL-positive cardiocytic and fibroblastic nuclei were readily recognized (TUNEL-immunocytochemistry, transmitted light, 150× magnification, *bar* represents 10 µm). M-3 (*down right*): TUNEL-positive cardiocytic and fibroblastic nuclei were prevalent (TUNEL-immunocytochemistry, transmitted light, 150× magnification, *bar* represents 10 µm). **b**. Percentage of TUNEL-positive nuclei per total nuclear area

**Fig. 4:**
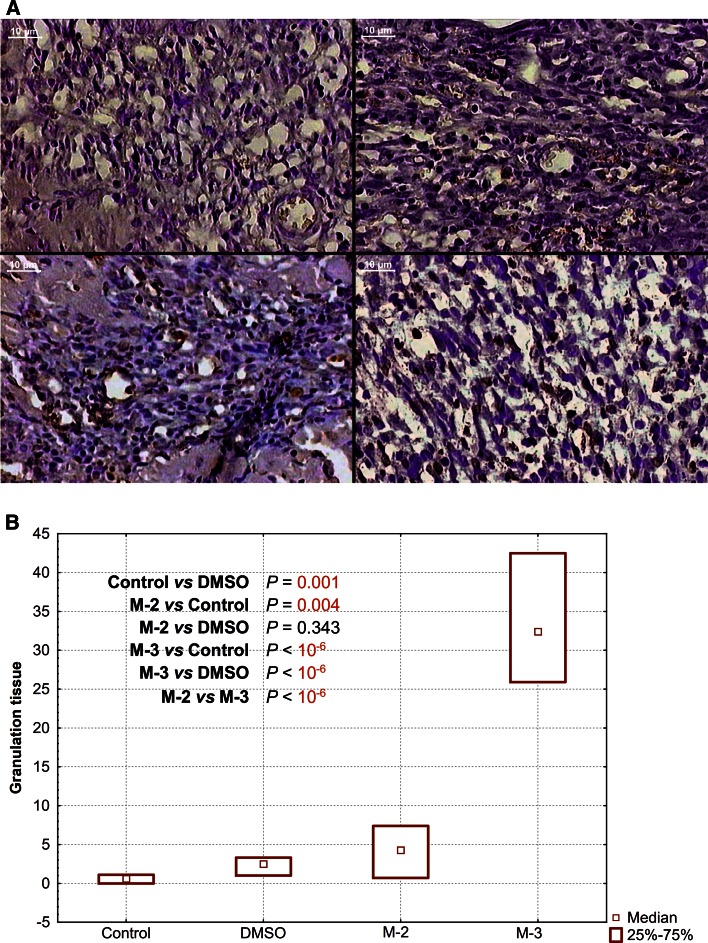
Representative images of apoptotic staining and their quantificative results in rats’ postischemic granulation tissue after single oral pretreatment with 20 mg kg^−1^ of M-2 or M-3. **a** TUNEL reaction. Control (*upper left*): All visible cells, including inflammatory infiltration, vessels and young elongated fibroblasts were TUNEL-negative (TUNEL-immunocytochemistry, transmitted light, 150× magnification, *bar* represents 10 µm). DMSO (*upper right*): Only few positively stained brown nuclei (TUNEL-positive) were visible inside dense inflammatory infiltration (TUNEL-immunocytochemistry, transmitted light, 150× magnification, *bar* represents 10 µm). M-2 (*down left*): TUNEL-positive endothelial cell nuclei and inflammatory cell nuclei were readily recognized (TUNEL-immunocytochemistry, transmitted light, 100× magnification, *bar* represents 10 µm). M-3 (*down right*): Numerous TUNEL-positive inflammatory and fibroblastic cell nuclei inside granulation (TUNEL-immunocytochemistry, transmitted light, 150× magnification, *bar* represents 10 µm). **b** Percentage of TUNEL-positive nuclei per total nuclear area

**Fig. 5:**
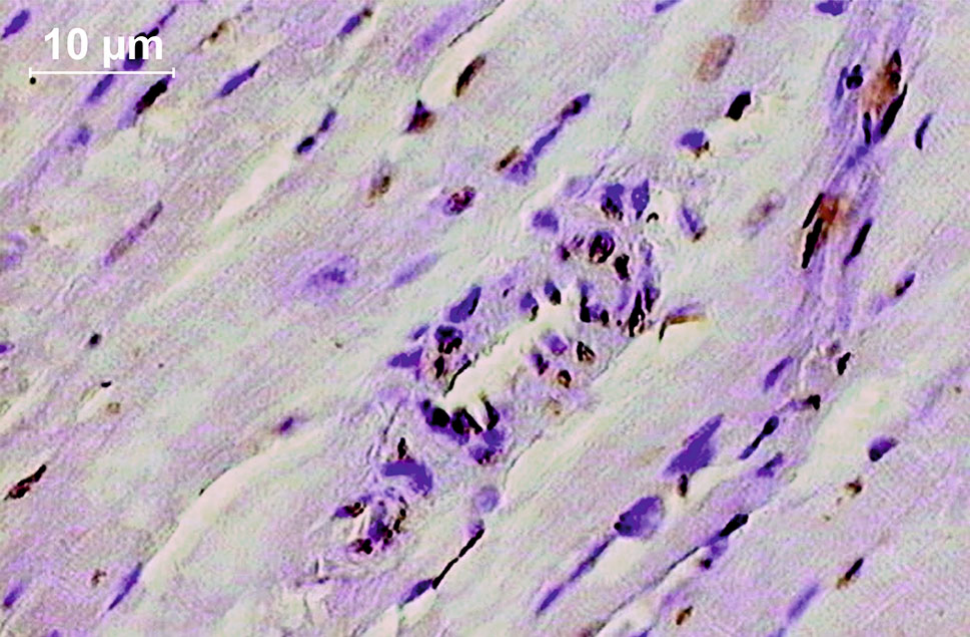
Numerous TUNEL-positive nuclei visible inside arteriolar wall and inside cardiomyocytes after single oral pretreatment with 20 mg kg^−1^ of M-2 in rats (TUNEL-immunocytochemistry, transmitted light, 200× magnification, *bar* represents 10 µm)

At 4th day after myocardial infarction, TUNEL-positive cell nuclei were present scanty in control and DMSO groups in myocardial cells as well as in granulation tissue, whereas in M-2 and M-3 groups brownish stained TUNEL- positive nuclei were easily recognized in both lesions (Figs. 3, 4). Moreover, in M-2 and M-3 treated animals, apoptotic cells were found also in arteriolar walls, whereas in control and DMSO groups this phenomenon was never exhibited (Fig. 5). Statistical analysis revealed marked increase in the amount of apoptotic nuclei after both furnidipines’ metabolites treatment in myocardial as well as granulation tissue, when compared to control and DMSO groups. Additionally, M-3 presented significantly stronger proapoptotic effect than M-2 in all cases. The statistical and quantitative results are presented in details in Figs. 3 and 4.

## Discussion

In chosen rats’ model of myocardial infarction followed by 15 min of reperfusion, the single oral pretreatment with both studied metabolites effectively reduced mortality index of the animals, did not markedly influence on blood pressure as well as on the ST-segment changes (Table 1, 2). Whereas the number of premature ventricular beats was significantly reduced in reperfusion in comparison to occlusion, this was found in all groups. Hence it could not be described as an effect of any of furnidpines’ metabolites.

It can be generally assumed that the all kinds of arrhythmias occurred in occlusion were strongly reduced in reperfusion. It is mainly due to the fact of enough long occlusion in this model, but not to the influence of tested agents. Nevertheless, it has been shown that the same dose of M-2 significantly reduced mortality as well as the ventricular fibrillation incidence and duration in reperfusion, but after just 7 min of LAD occlusion in the model of ischemia- and reperfusion arrhythmias in rats [35]. The biochemical findings in blood at 4th day after myocardial infarction showed that only the M-3 pretreatment reduced significantly GOT concentration in comparison to control as well as it strongly reduced CK values in comparison to M-2 and DMSO groups (*p* < 0.05). Although some beneficial effects of the tested compounds were present, in view of our previous experiments with these agents [34, 35, 38, 57] it could be concluded that the model used in present study is not suitable to quantify their optimal cardioprotective effects.

Despite the fact that no visible differences were found in routine histopathological investigation, the most intriguing results of this research concern the programmed cell death study. At 4th day after reperfused infarcted rats’ heart, TUNEL-positive cell nuclei were present scanty in myocardial cells as well as in granulation tissue in control and DMSO groups, whereas in M-2 and M-3 groups brownish stained TUNEL-positive nuclei were easily recognized in both tissues. At least in this issue, the clear difference between M-3 and M-2 due to proapoptotic influence was found. In both tissues, the M-3 increased approx. 30 % the number of TUNEL-positive nuclei, while the M-2—approx. 5 %. Moreover, several differences in apoptotic rate between ischemic myocardium and granulation tissue were noticed after application of each agent. Percentage of the total effect in both tissues (calculated as in the example of DMSO granulation: 5/negative/+3/positive/ = 8, what results 3/positive/is 37 % of total) revealed that: (a) DMSO stimulated apoptosis in granulation tissue only (37 %), (b) M-2 is more powerful in apoptosis stimulation in granulation tissue than in post-ischemic myocardium cells (52 vs 32 %), (c) M-3 is equally potent in apoptosis stimulation in both tissues (approx. 50 %).

According to these results, M-2 is more likely in promoting apoptosis in granulation tissue, while M-3 possess more balanced profile for apoptosis stimulation in both tissues.

It should be also noticed that we cannot exclude the DMSO membrane effects on apoptosis stimulation in granulation tissue caused by both furnidipines’ metabolites. The obligatory use of DMSO as a solvent for dihydropyridines derivatives (even in the concentrations lower than 0.4 %) will always mimics to some extend these agents’ action due to its ability to generate apoptosis *per se* [58, 59].

Interestingly, apoptotic cells were also found in arteriolar walls after both metabolites administration, whereas in control and DMSO groups this phenomenon was not observed at whole.

In view of the fact presented above, the fundamental, controversial questions return: whether the apoptosis in general is beneficial for healing, remodeling processes after myocardial infarction and whether the proapoptotic or antiapoptotic agents are wanted participants in this game?

Although programmed cell death proceeds by the same mechanism in each cell, the meaning of this process in heart healing can be different, due to the consequences it brings. Whereas ongoing process of apoptosis may be beneficial in one condition, in other it may be linked with detrimental effects. It appears obvious that apoptosis contributed with myocyte loss as well as organising fibrotic tissue in the place of ischemia results in sustained contractile failure of myocardium [60]. Other authors reported also that myocytes apoptosis is likely to precede necrosis connected with disintegration of cells and in consequence, deterioration of heart function [61, 62]. Furthermore, the experimental study on mice proved that inhibition of apoptotic cascade by transfer of adenoviral antiapoptopic soluble Fas gene in the 3rd day after ligation-induced myocardium infarction could be potentially valuable therapeutic strategy in cardiac diseases [63]. In addition, recent data revealed as well that anoikis, defined as special type of PCD induced by cell detachment, is responsible for pathological remodeling of cardiovascular tissue in heart failure [64, 65].

On the other hand, the functional role of apoptosis in granulation tissue seems to have different meaning. According to the fact that TUNEL detection of apoptosis is not limited to the dying myocytes, but it also points out other cells undergoing this process [62], TUNEL positivity in granulation tissue may be related to the presence of inflammatory cells which accumulate in the injured myocardium area. It is well established the infiltrated leucocytes and macrophages play essential role in infarct healing by their ability of scavenging ischemic area from death myocytes as well as stimulation of angiogenesis and myofibroblast proliferation [66]. In addition, the inflammatory cells after fulfilling their role undergo the process of PCD which is, in comparison to necrosis, a ‘safe death pathway’ which protects survived myocytes from excessive inflammation process caused by release of their cytotoxic factors such as cytokines and proteases [62].

Moreover, our finding of apoptotic cells in arteriolar walls in M-2 and M-3 treated groups sounds favourably in the light of the statement indicating that apoptosis helps in rebuilding the intima of coronary vasculatures by elimination of injured cells, what results in quicker growth of new endothelium [62].

In general, the balance between programmed cell death and regeneration processes in all kinds of tissues seems to be crucial aspect in determination of myocardium recovery after infarction. In the light of the histological results presented above, M-2 shows itself as more attractive agent for oral pretreatment in early stages of ischemia by non-stable individuals suggested elsewhere [57] due to its more specific action in stimulation resorptive processes in granulation tissue as well as in arteriolar walls. Furthermore, this working hypothesis enlarge our outcomes from previous study where M-2 positively influenced on post-infarction heart remodeling. We have proved, M-2 administration from 6th to 35th day after infarction effectively prevents cardiomyopathy development through the revitalisation of the coronary arteries, infarct scar remodeling as well as acceleration of angiogenic events [38].

Although apoptosis can be induced by ischemia itself [62], it cannot succeed without oxygen as it is active, energy- requiring process. Accordingly, reperfusion is one of the triggers responsible for promoting the apoptotic cascade and is associated with higher TUNEL positivity [62, 67]. It has been proven the apoptotic nuclei are particularly evident in the infarcted areas where spontaneous reperfusion most often occurs [61, 68]. The higher amount of TUNEL-positive nuclei in samples collected from M-2 and M-3 groups suggests better reperfusion in myocardium cells in groups pretreated with furnidipines’ metabolites in contrast to control and DMSO groups. Clearly, this effect may be achieved due to their proven various vasodilatatory effect as well as increase in cardiac coronary flow [34].

In order to explain relationship between histological outcomes of presented study evoked by furnidipines’ metabolites we should also take a closer look at the molecular mechanism of apoptosis and M-2 actions in general. It is well known that myocardial ischemia–reperfusion is associated with elevated content of intracellular Ca^2+^ (especially in mitochondria), which is also a major trigger of the apoptotic intrinsic pathway [69]. Due to the fact all dihydropyridines are proved to possess the ability of L-type calcium (Ca) channel blocking, they seem to be potential pharmacological protectors of the damaging caused by pro-apoptotic intracellular Ca^2+^ overload. Since many contradictory opinions were presented on this issue, their role in this application still remains controversial. Some authors claim that these group of drugs cannot be effective in apoptosis inhibition while they do not influence on the sodium-hydrogen exchanger (NHE) which is indicated to be major Ca^2+^ transporter in the process of cell death [62, 69]. It has been suggested M-2 acts as a sodium and outward potassium ATP-dependent channels gating protector, while it effectively reduced (>90 %) anoxia-induced action potential shortening and veratridine-induced action potential lengthening [35]. Despite of the lack of its cardio-depressive action [34] which could be linked with the M-2 poor activity on calcium channels, some experiments have revealed decrease of the intracellular free calcium ion concentration during hypoxia in non-stimulated isolated guinea pig cardiomyocytes after M-2 administration. This could be due to an effect on an alternative calcium entry via modified sodium channels or via sodium/calcium ions exchange [31]. It is worth stressing that besides its wide spread activity, M-2 as a potential NO donor is supposed to decrease intracellular Ca concentration through cyclic GMP-dependent mechanism [38, 62]. Nevertheless, it should be remembered that the crosstalk between mechanism of apoptosis and M-2 action is suggestive and needs further elucidation.

In conclusion, searching for confirmation of results obtained in our previous studies concerning favourable effects of furnidipines’ metabolites (especially M-2) administration on mortality, blood pressure, arrhythmias and post-infarcted remodeling, one could consider that our expectations for next beneficial outcomes to some extent failed. Despite there are no distinguishing differences regarding FUR metabolites hemodynamic profiles as well as arrhythmias incidence and duration, fortunately, we decided to challenge the problem at the histopatological level as well. Surprising effect of M-2 and M-3 single pretreatment paired with higher apoptotic rate in post-ischemic myocardium as well as in granulation tissue and what is more, in arteriolar walls is a major interest and again makes furnidipines’ metabolites intriguing objects for future investigations.

It must be remembered, M-2 and M-3 are common metabolites present in degradation pathways of many and widely used dihydropyridines in clinic, especially in long-term secondary prevention of myocardial infarct.

This key fact put the new outlook on understanding additional mechanism and effects of not only furnidipines’ metabolites as well as other dihydropyridines.

Taking into account, that programmed cell death could play positive role in post-infarcted heart healing, it might be summarized: M-2 is more specific in stimulation of repairing processes in granulation tissue after occlusion-induced myocardium infarction. Accordingly, it may allow to introduce a novel class of agents with attractive properties, which do not belong to dihydropyridines derivatives due to their different chemical structure.
